# Newly discovered KI, WU, and Merkel cell polyomaviruses: No evidence of mother-to-fetus transmission

**DOI:** 10.1186/1743-422X-7-251

**Published:** 2010-09-22

**Authors:** Mohammadreza Sadeghi, Anita Riipinen, Elina Väisänen, Tingting Chen, Kalle Kantola, Heljä-Marja Surcel, Riitta Karikoski, Helena Taskinen, Maria Söderlund-Venermo, Klaus Hedman

**Affiliations:** 1 Department of Virology, Haartman Institute, University of Helsinki, BOX 21, FIN-00014, Helsinki, Finland; 2 Centre for Expertise for Health and Work Ability, Finnish Institute of Occupational Health, Topeliuksenkatu 41 a A, FIN-00250, Helsinki, Finland; 3 National Institute for Health and Welfare, P.O. Box 301, FIN-90101, Oulu, Finland; 4 Department of Pathology, Helsinki University Central Hospital Laboratory Division, Haartmaninkatu 3, FIN 00290, Helsinki, Finland; 5 Department of Public Health, University of Helsinki, BOX 21, FIN-00014, Helsinki, Finland; 6 Department of Virology, Helsinki University Central Hospital Laboratory Division, Haartmaninkatu 3, FIN 00290, Helsinki, Finland

## Abstract

**Background:**

Three* human polyomaviruses have been discovered recently, KIPyV, WUPyV and MCPyV. These viruses appear to circulate ubiquitously; however, their clinical significance beyond Merkel cell carcinoma is almost completely unknown. In particular, nothing is known about their preponderance in vertical transmission. The aim of this study was to investigate the frequency of fetal infections by these viruses. We sought the three by PCR, and MCPyV also by real-time quantitative PCR (qPCR), from 535 fetal autopsy samples (heart, liver, placenta) from intrauterine fetal deaths (IUFDs) (N = 169), miscarriages (120) or induced abortions (246). We also measured the MCPyV IgG antibodies in the corresponding maternal sera (N = 462) mostly from the first trimester.

**Results:**

No sample showed KIPyV or WUPyV DNA. Interestingly, one placenta was reproducibly PCR positive for MCPyV. Among the 462 corresponding pregnant women, 212 (45.9%) were MCPyV IgG seropositive.

**Conclusions:**

Our data suggest that none of the three emerging polyomaviruses often cause miscarriages or IUFDs, nor are they transmitted to fetuses. Yet, more than half the expectant mothers were susceptible to infection by the MCPyV.

## Background

Among the five* human polymaviruses known, aside from the BK virus (BKV) and JC virus (JCV) [1,2], three* new ones, KI polyomavirus (KIPyV), WU polyomavirus (WUPyV), and Merkel cell polyomavirus (MCPyV) have been discovered during the past few years by use of advanced molecular techniques [3-5]. In their DNA sequences, KIPyV and WUPyV are interrelated more than MCPyV, which differs from all the human polyomaviruses known [6]. The KIPyV and WUPyV were discovered in nasopharyngeal aspirates (NPA) from children with respiratory tract infections [3,4]. Although many reports have confirmed their presence in the upper airways of patients with respiratory illness, evidence is lacking of their pathogenicity in this context [7-10,12]. For these viruses, their tropism and clinical significance are unknown. Likewise, for the tumorigenic MCPyV [5], also found in the nasopharynx [13-15], the mode of transmission and, host cells, as well as latency characteristics are yet to be established. MCPyV DNA has been detected in a variety of specimen types including skin, saliva, gut, and respiratory secretion samples [5,16,17]. Recovery of complete MCPyV genomes from the skin of 40% of healthy adults and PCR detection of MCPyV in the skin of almost all adults by cutaneous swabbing suggests that most adults are persistently infected with this polyomavirus [18]. Another recent study revealed the viral DNA in environmental samples (sewage and river water) [19], confirming that MCPyV indeed is a ubiquitous virus.

Serological studies have shown that initial exposure to KIPyV and WUPyV, as well as MCPyV occurs often in childhood, similar to that for BKV and JCV, and that MCPyV circulates widely in the human population [20-24]. Although most adults have been exposed to MCPyV, the exact site(s) of MCPyV infection remain unclear. Vertical transmission of many human DNA and RNA viruses is well established. However, for *Polyomaviridae *this mode of transmission is far from clear. Transplacental transmission of BKV was first suggested by detection of the virus DNA in fetal tissues [25], while others obtained no evidence of vertical transmission [26]. IgM studies of BKV and JCV in cord blood samples showed no apparent association with congenital infection [27,28].

These findings prompted us to investigate a sizeable material of fetal autopsy samples (placenta, heart, liver) for the presence of the three polyomaviruses in order to determine whether these viruses give rise to fetal infections. We also studied the corresponding maternal sera for MCPyV IgG antibodies, by a newly established virus like particle (VLP) - based IgG assay (Chen et al, in revision).

## Materials and methods

### Clinical samples

The DNA studies were carried out using formalin-fixed, paraffin-embedded (FFPE) tissues - placenta, heart, and liver - after intrauterine fetal deaths (IUFDs, N = 169), miscarriages (N = 120), or using as controls, specimens from induced abortions (N = 246) performed exclusively due to medical indications. From each fetus, 3 organs (when available; placenta, heart, and liver) were initially studied in pools, by PCR for the three polyomaviruses (KI, WU, and MC). In the positive pool its constituent tissues (placenta and heart) were then re-examined separately. Thus, a total of 535 fetuses were included in the overall cohort. The sampling in the Helsinki region occurred from July 1992 to December 1995 and January 2003 to December 2005 [29]. The gestational weeks of the fetal deaths ranged from 11 to 42. In this study, IUFD corresponds to fetal loss having occurred during or after the 22nd gestational week, and miscarriage to fetal loss having occurred earlier. Our number of IUFDs represents 58% of all occurring in Helsinki during the study period.

We furthermore examined for the presence of MCPyV IgG antibodies all serum samples available from the corresponding mothers (n = 462). These samples had been collected at the municipal maternity centers during antenatal screening around the 9th gestational week (mean 9; median 9; range 2 to 36) and were stored frozen at the Finnish Maternity Cohort, National Institute for Health and Welfare, Oulu, Finland. The mothers' ages ranged between 18 and 45 years (mean 31, median 31).

The study was approved by the Coordinating Ethics Committee of the Hospital District of Helsinki and Uusimaa. Permission for use of fetal tissues was obtained from the National Supervisory Authority for Welfare and Health (Valvira).

### DNA preparation and PCRs

The paraffin blocks were punch-biopsied, proteinase K-digested, and the DNA was prepared by salting out, as described [29]. Briefly, tissue lysates were heated at 95°C for 10 min. The paraffin then appeared floating on the surface, after centrifugation at 13,200 rpm for 5 minutes (Eppendorf; 4°C). Sodium chloride was added to achieve a final concentration of 1.2 mol/L, and the sample was mixed for 20 s and recentrifuged. The supernatant was transferred to a new tube, carefully avoiding particles. The DNA in the supernatant was precipitated with absolute ethanol and was redissolved in 60 mL of water. The DNA solution diluted 1 to 10 was stored at - 20°C until use. Water, as a negative control, was inserted between every 20 samples and was prepared along with the tissue pools.

All these DNA preparations were *β *-globin-PCR-positive, pointing to DNA stability and lack of appreciable PCR inhibition. The WUPyV and KIPyV nested PCRs employed primer set A (table 1) [12]. For MCPyV DNA detection by qualitative PCRs, two primer sets were used (table 1); all samples were first studied by the LT3 nested PCR [15], and were reanalyzed by the LT1/M1 nested PCR [30], and samples with positive results by agarose gel electrophoresis were DNA sequenced. As short PCR products ought to be used when working with FFPE tissues [31,32] we reanalyzed all pools with a real-time quantitative PCR with an amplicon length of 59 bp, as described [13].

**Table 1:** Primers and probe used to detect KIPyV, WUPy and MCPyV.

PCR and Virus	Primer Sequence 5 → 3		Region	Amplicon size
	**Forward**	**Reverse**		

**Nested PCR outer primers**				
KIPyV, WUPyV	ATCTRTAGCTGGAGGAGCAGAG	CCYTGGGGATTGTATCCTGMGG	VP2	336 bp
MCPyV (LT3)	TTGTCTCGCCAGCATTGTAG	ATATAGGGGCCTCGTCAACC	LT	309 bp
MCPyV (LT1)	TACAAGCACTCCACCAAAGC	TCCAATTACAGCTGGCCTCT	LT	440 bp
**Nested PCR inner primers**				
KIPyV, WUPyV	RTCAATTGCTGGWTCTGGAGCTGC	TCCACTTGSACTTCCTGTTGGG	VP2	277 bp
MCPyV (LT3)	TGACGTGGGGAGAGTGTTTTTG	GAGGAAGGAAGTAGGAGTCTAGAAAAG	LT	155 bp
MCPyV (M1)	GGCATGCCTGTGAATTAGGA	TTGCAGTAATTTGTAAGGGGACT	LT	179 bp
**MCPyV qPCR primers**	TGCCTCCCACATCTGCAAT	GTGTCTCTGCCAATGCTAAATGA	VP1	59 bp
MCPyV probe	FAM-TGTCACAGGTAATATC-MGBNFQ			

### PCR sensitivity

For detection of KIPyV/WUPyV and MCPyV we performed a highly sensitive PCR assay [15]. As positive controls and to determine assay sensitivities by limiting dilution analysis, plasmids containing the VP2 gene of WUPyV (EU693907) and KIPyV (EU358767) and the LT3 amplification product of MCPyV (EU375803) were constructed; the amplification products of the VP2 genes were cloned into pCR8/GW/TOPO (Invitrogen; Carlsbad, CA, USA) whereas the MCPyV LT3 region was synthesized and cloned into pGOv4 by Gene Oracle, Inc. (San Leandro, USA). In the MCPyV and KIPyV/WUPyV assays, plasmid controls with 30 and 5 copies/reaction were reproducibly positive, respectively. Of note, the sensitivities were unaffected by the inclusion of genomic human DNA from cultured 293T cells at 100 ng per reaction (4 ng/*μ*l). In non-nested format with 40 PCR cycles the LT3 primers had a sensitivity 1 log lower than that of the nested assay both in the presence and absence of human genomic DNA. In addition, we also used a previously established real time quantitative PCR for MCPyV, where a plasmid control with 2 copies/reaction was reproducibly positive [13].

### Sequence analyses

The MCPyV PCR products were purified for automated sequencing using the High Pure PCR product purification kit (Roche). The resulting DNA sequences using BLAST were aligned against the reference sequences in GenBank including accession numbers of [gb|EU375803.1]; [gb|EU375804.1]; [gb|FJ173815.1]; [gb|FJ464337.1]; [gb|HM011538] and [gb|HM011557].

### MCPyV antibody EIA

MCPyV IgG antibodies were measured by EIA based on virus protein 1 (VP1) VLPs (Chen et al, in revision). Briefly, the VP1-VLPs expressed in insect cells and purified by CsCl density gradient centrifugation were biotinylated and attached (at 60 ng/well) to streptavidin-coated microtiter plates (Thermo Scientific) and saturated with a sample diluent (Ani Labsystems). The sera (1:200) were applied in duplicate, the bound IgG were quantified with peroxidase-conjugated anti-human IgG (1:2000; DakoCytomation) using H_2_O_2 _and OPD substrates, and the absorbances at 492 nm were read after blank subtraction. The EIA cut-off for IgG positivity is 0.120 OD units.

## Results and Discussion

Among the 535 fetal autopsy samples studied in pools, none was PCR positive for KIPyV or WUPyV DNA. On the other hand, one pool was positive for MCPyV by the LT3 PCR. Tissue samples from 2 sites were available for further study of this fetus. On retesting of the placenta and fetal heart separately, the heart was PCR negative and the placenta was positive for MCPyV. However, it was negative by the LT1/M1 PCR for MCPyV. DNA preparations re-extracted from the other half of the same punch biopsy, as well as those obtained via another punch from the same paraffin block, showed exactly the same MCPyV DNA results. Furthermore, we studied all samples with a real-time qPCR for MCPyV of a different genomic region, with an identical result; the placenta was positive with a Ct value of 38.4, while the biopsy from the fetal heart was negative. To verify the specificity of the amplified products and to detect possible genomic variants, the MCPyV LT3 PCR products were sequenced. They showed 100% homology with all the existing database sequences. N.B., the miscarried fetus, with gastroschisis and umbilical cord complications, was deceased in 1994, in gestational week 17.

In addition, we examined for circulating MCPyV IgG antibodies the corresponding pregnant women. Of the 462 maternal sera, 45.9% (212) showed positive results.

We searched formalin-fixed, paraffin-embedded tissues - placenta, heart, and liver - of 535 fetal autopsy samples for the KI, WU, and MC polyomaviruses. We found no genomic DNAs of KIPyV or WUPyV in any of the stillborn or deceased fetuses. This suggests that during the study period neither of these two newly found viruses (i) often caused miscarriages or IUFDs, (ii) nor were incidentally (as bystanders) transmitted to remain in the fetuses succumbing for other reasons. Whether the exclusive mechanism in our mid-size series was the serendipitous absence of maternal infections (primary; secondary) caused by these viruses, or pathogenetic resistance by other mechanisms, remains to be shown. On the other hand, it was interesting to observe LT3 PCR and VP1 qPCR positivity for MCPyV (reproducibly, and of correct DNA sequence) in the placenta of one miscarriage in the 17th gestational week. The negativity of this placenta with the other MCPyV PCR (LT1/M1) may be due to the known sensitivity difference of the PCR assays [15,30].

As for the previously known human polyomaviruses BKV and JCV, no fetal autopsy materials have been found positive for JCV DNA, whereas one study [25] reported a high genoprevalence of BKV DNA; BKV vertical transmission has been denied by others [11,33,34], however.

Ours is to our knowledge the first study in which fetal tissues have been searched for the newly discovered human polyomaviruses. Serology has shown that a high proportion of adults have been exposed to MCPyV, and that the infection can be acquired early in life [20-24]. We detected an IgG seroprevalence for MCPyV of 45.9% among the pregnant women. This value is in line with previous reports and shows that more than half our women were susceptible to MCPyV infection. Tolstov et al. studying serologically 6 children 1 year or younger found no evidence of MCPyV vertical transmission [24].

## Conclusions

While the three* emerging polyomaviruses occur frequently in tissues of many different types, and MCPyV also in environmental samples [3,4,13,15,18,35], our PCR data from 535 pregnancies suggest that none of these viruses are frequently transmitted vertically. Further studies with larger populations may, however, be warranted to determine which role, if any, MCPyV plays in pregnant women and their offspring.

## Competing interests

The authors declare that they have no competing interests.

## Authors' contributions

MS carried out the PCR and serological assays, analyzed the data, and participated in writing. AR and EV organized the clinical materials and carried out the DNA extractions. TC accounted for the serology part. KK participated in the methods design and setup. HMS, RK, and HT collected the specimens and contributed to the data analysis. MSV and KH conceived the study, participated in its design and coordination and accounted for the manuscript writing. All authors read and approved the final manuscript.
